# Fish as Food

**Published:** 1874-12

**Authors:** 


### FISH AS FOOD.

Some economic writers maintain that fish has no food-value worth speaking of ; others say that fish-food must occupy a middle position between vegetables and beef and mutton. Again, a learned authority says that fish, well cooked, with oil or fat of some kind, or served with butter when brought to table, "is chemically the same as meat, so far as nutrition is concerned." Another writer says that fish as food is only fit for children and invalids, and is totally unfitted to support the health and vigor of men or women engaged in laborious occupations. As usual in such disputes, we may hold that the truth lies between the two extremes. Many people following laborious occupations live largely upon fish. Millions of human beings depend upon fish almost exclusively as an article of diet. In China and Japan it is universally employed to the exclusion of meat. The inhabitants of all sea coasts use this food largely, and are not lacking in vigor and strength, although depending upon fish for centuries.

In Portugal, fish fried in oil forms a very large proportion of the food of the population ; their fish diet is supplemented by a little bread and fruit, and although the peasantry of the land never partake of flesh meat they are a hardy, vigorous, and brave people.

Fish abounds in phosphorous, and is therefore considered as most valuable brain food. The more oily varieties form a very concentrated article of diet, capable of sustaining life even when consumed in small quantities ; and, if agreeable to the stomach, will supply all the elements necessary to the support of vigorous health.

